# Draft Whole-Genome Sequences of 10 *Aeromonas* Strains from Clinical and Environmental Sources

**DOI:** 10.1128/MRA.00170-19

**Published:** 2019-07-25

**Authors:** Katie Gray, Luke R. Green, Roy R. Chaudhuri, Jonathan G. Shaw

**Affiliations:** a Department of Infection, Immunity and Cardiovascular Disease, University of Sheffield, Sheffield, United Kingdom; b Department of Molecular Biology and Biotechnology, University of Sheffield, Sheffield, United Kingdom; University of Maryland School of Medicine

## Abstract

*Aeromonas* bacteria are able to cause disease in a wide range of animals from humans to fish. In this article, we report the draft whole-genome sequences of 10 *Aeromonas* strains from clinical and environmental sources. These genome sequences will provide a repository of information for further investigations into the pathogenicity of this enigmatic pathogen.

## ANNOUNCEMENT

*Aeromonas* species are aquatic bacteria found in brackish water and freshwater and are able to cause disease in both cold-blooded and warm-blooded animals (1). In humans, they can give rise to numerous clinical manifestations but are mostly associated with gastrointestinal disease and wound infections (2). A plethora of factors have been linked with aeromonad pathogenicity, including toxigenic factors, such as aerolysin and type III secretion systems (T3SS), and adhesins, such as type IV pili and both polar and lateral flagella (3–5). However, aeromonad pathogenicity is thought to be multifactorial and not reliant upon one virulence determinant. Aeromonas caviae strains Sch3 and Sch29 were isolated from children presenting with gastroenteritis at Sheffield Children's Hospital, and Aeromonas veronii biovar sobria BC88 was isolated from a child with dysentery in Western Australia (6); all the other aeromonads were environmental strains isolated from the River Don at various locations within South Yorkshire, United Kingdom. *A. caviae* Sch3 has been extensively studied due to its production of lateral flagella (4) and its ability to glycosylate its polar flagella (7, 8). *A. veronii* biovar sobria BC88 is a model for aeromonad adhesion, as it produces a type IV bundle-forming pilus (5). The analysis of the genomes presented here will allow a deeper understanding of the biology of aeromonads in relation to both their physiology and pathogenicity.

All environmental strains were isolated from river water by inoculation onto cystine lactose electrolyte-deficient (CLED) agar plus Andrade’s indicator (Oxoid) and Brilliance UTI clarity agar (Oxoid), as these were nonselective and provided a differential presumptive identification of organisms. Human clinical strains were isolated by the inoculation of feces onto *Aeromonas* isolation agar (Fluka) containing ampicillin. All strains were identified to the species level by multisequence alignment against the sequences of known aeromonad species. All strains were grown in tryptic soy broth (TSB), and genomic DNA was extracted using a QIAamp DNA minikit (Qiagen). For each of the 10 *Aeromonas* strains, genomic DNA libraries were prepared using the Nextera XT library kit (Illumina, San Diego, CA). The genomes were nucleotide sequenced using an Illumina HiSeq 2500 platform at MicrobesNG (University of Birmingham, UK). Sequencing used 2 × 250-bp paired-end reads that gave from 41 to 150× depth (Table 1). Trimmomatic v0.38 (9) was used to trim the reads with a sliding window quality cutoff of Q15, and read quality analyses were performed with FastQC software v0.11.8 (http://www.bioinformatics.babraham.ac.uk/projects/fastqc/). The genomes were assembled and annotated using SPAdes v3.7 (10) and Prokka v1.12, respectively, using the standard default settings (11). Genome assembly metrics were calculated with QUAST, and the number of annotated coding sequences (CDS) for each aeromonad genome is shown in Table 1.

**TABLE 1:** Genome accession numbers and characteristics of 10 *Aeromonas* strains taken from either a clinical or environmental source

Strain	Species (source)	Mean coverage (×)	No. of reads	No. of contigs	Largest contig (bp)	Total length (bp)	GC content (%)	*N*_50_ (bp)	*N*_75_ (bp)	*L*_50_ (contigs)	*L*_75_ (contigs)	No. of CDS	ENA accession and assembly no.
Sch29	*A. caviae* (human clinical diarrhea)	49.94	874,346	195	182,866	4,425,373	61.34	86,948	50,669	21	37	3,953	ERS3090042, GCA_901202955
											
Sch3N	*A. caviae* (human clinical diarrhea)	84.83	1,155,915	192	240,555	4,737,922	61.39	90,385	43,401	18	37	4,313	ERS3090043, GCA_901212305
											
BC88	*A. veronii* (human clinical dysentery)	111.91	1,381,586	155	1,023,078	4,604,788	58.54	215,763	118,836	6	13	4,065	ERS3090044, GCA_901212295
											
KLG1	A. hydrophila (environmental water)	53.88	730,744	154	260,972	4,908,673	61.22	103,738	64,788	15	30	4,373	ERS3090045, GCA_901212375
											
KLG2	A. allosaccharophila (environmental water)	150.39	1,670,538	85	626,394	4,513,594	58.87	292,420	141,273	5	11	4,059	ERS3090046, GCA_901212385
											
KLG5	*A. veronii* (environmental water)	70.63	974,322	103	818,983	4,740,061	58.51	280,270	130,857	6	12	4,269	ERS3090047, GCA_901212355
											
KLG6	A. media (environmental water)	104.31	1,255,304	454	159,134	4,542,840	61.20	36,002	19,277	40	80	4,070	ERS3090048, GCA_901212365
											
KLG7	*A. veronii* (environmental water)	53.29	687,242	104	345,454	4,552,893	58.80	139,212	84,196	11	21	4,069	ERS3090049, GCA_901212345
											
KLG8	*A. veronii* (environmental water)	54.65	738,573	76	568,763	4,590,381	58.62	198,583	115,053	7	15	4,137	ERS3090050, GCA_901212395
											
KLG9	*A. veronii* (environmental water)	40.98	578,468	74	399,442	4,605,689	58.71	180,084	120,803	9	17	4,160	ERS3090051, GCA_901212405
											

The sequences will provide a great resource for further investigations into the physiology and pathogenicity of the *Aeromonas* genus.

### Data availability.

The reads used for assembly of the 10 annotated aeromonad genomes were deposited in the European Nucleotide Archive (ENA) at the European Molecular Biology Laboratory (EMBL) under the accession number PRJEB31025. The specific accession numbers for each sample are supplied in Table 1. The versions described in this paper are the first versions.
